# Swine welfare perceptions among future animal professionals in Latin America

**DOI:** 10.3389/fvets.2026.1874259

**Published:** 2026-07-23

**Authors:** Gustavo Donoso, Andreia G. Arruda, Leonardo Klüppel, Juliana Bonin Ferreira, Monique Pairis-Garcia

**Affiliations:** 1 Department of Population Health and Pathobiology, College of Veterinary Medicine, North Carolina State University, Raleigh, NC, United States; 2 Department of Veterinary Preventive Medicine, College of Veterinary Medicine, The Ohio State University, Columbus, OH, United States; 3 Department of Management and Human Resources, Max M. Fisher College of Business, The Ohio State University, Columbus, OH, United States

**Keywords:** animal welfare education, curriculum, Latin America, swine welfare, teaching

## Abstract

The importance of swine welfare has grown in Latin America, driving universities in this region to place significant efforts in developing educational opportunities to better prepare future animal professionals. While students' perspectives are central to assess the efficacy of these programs, research remains limited. To tackle this issue, this study aimed to explore, through an online survey, Latin American future animal professionals' perceptions and attitudes toward swine welfare and examine their confidence in discussing and addressing welfare issues specific to pigs. The survey was distributed among universities located in Ecuador, Brazil, Chile, and Honduras to undergraduate and graduate students in veterinary and animal science programs. Participant responses (*n* = 216) were analyzed using cluster and risk factor analysis. Cluster analysis revealed three distinct groups, all of which agreed on the importance of animal welfare but varied in how students value swine welfare education and in their confidence to communicate and address swine welfare issues. Additionally, risk factor analysis revealed that students' source of information was an important cluster membership predictor. Overall, this study provided insights into students' perspectives, values, and confidence regarding swine welfare, as well as recommendations for future research that universities and researchers should consider to further enhance the education of future animal professionals in the region and beyond.

## 1 Introduction

Animal welfare science in education is one of the recognized core competencies required to successfully prepare students for careers in animal health and production (1–5). Animal welfare knowledge for such professionals has been incorporated as a top priority by the World Organization for Animal Health (WOAH), given the impact that such training and knowledge can have on improving the lives of animals under human care (6). Particularly, animal welfare education is important for animal professionals working with pigs, as they serve a direct role in safeguarding animal welfare by responsibly optimizing swine health, behavior, and productivity (7–10).

In Latin America, there is evidence demonstrating a growing interest toward farm animal welfare (11) and particularly its application in swine production (12). In this context, animal welfare is also gradually gaining importance within veterinary medicine and animal science education in this region (13). Veterinary medicine and animal science programs in Ecuador, Brazil, Honduras, and Chile have put significant resources into expanding educational opportunities in animal welfare training, especially regarding farm animal welfare (14–16). Accordingly, swine welfare has been established as an important component of the academic training of these programs, with proposed coursework increasingly integrating pig welfare principles and ethical aspects of pig production (17).

However, despite recent advancement in curriculum development and interest in animal and swine welfare education amongst Latin American universities, limited studies to date have explored perceptions and attitudes of Latin American students within the animal profession (18). Some studies do exist exploring certain concepts in animal welfare science, including work conducted in Costa Rica (19), Mexico (20), Brazil (21), Chile (22), and Colombia (23). However, all of these literature have centered on broad general perceptions of welfare or have focused on cattle welfare in production and welfare aspects related to forensic science. To date, no studies have specifically addressed swine welfare perspectives from Latin American future animal professionals and several countries in the region remain unexplored. Such information can provide feedback on how current educational programs are expanding students' animal welfare knowledge and highlight any gaps that currently exist in swine welfare education in this region. Therefore, the objectives of this study were to explore Latin American future animal professionals' perceptions and attitudes toward swine welfare and explore their confidence in discussing and addressing welfare issues specific to pigs.

## 2 Materials and methods

### 2.1 Ethical statement

This study was reviewed and deemed exempt by the Institutional Review Board at North Carolina State University (Protocol #27685) prior to the initiation of data collection.

### 2.2 Study population and Survey Development

An online survey targeting undergraduate and graduate students enrolled in animal science, veterinary and other animal-related programs was developed using Qualtrics survey software (Qualtrics, Provo, UT, USA). The online survey link was distributed via email through a listserv of 60 contacts across 33 different institutions in Ecuador, Brazil, Chile, and Honduras to professors and administrators in veterinary and animal science programs. These countries were chosen based on the professional network of involved collaborators. In the email, researchers asked professors and administrators to share the survey with undergraduate and graduate students within their departments and with other relevant colleagues to further expand the sample size.

The survey remained open for 8 weeks and was offered both in Spanish and Portuguese based on the targeted countries. The Spanish version was distributed in Ecuador, Chile, and Honduras between late February and mid-April 2025, while the Portuguese version for participants located in Brazil, was available from late March to late May 2025. Prior to being able to access the survey, participants were required to accept and give consent, authorizing the use of their anonymous responses and demographic information. No incentives were offered and participants were free to skip any questions they did not feel comfortable with or did not want to answer.

The survey was divided into three sections and contained a variety of question types, including multiple choice, Likert scale, and a matrix table. The questions for this survey were adapted from previous published research on animal welfare perspectives among animal science and veterinary student populations (2, 24). The first section focused on students' personal experiences with swine and consisted of multiple-choice questions (Table 1). The second section, composed of Likert scale questions, explored the importance students placed on animal and swine welfare topics and their perceived level of confidence in each area (Table 1). In addition, a matrix table question was also included in section two where students rated the importance placed on health, production, behavior, pain, emotional states and ethics/societal concerns for pig welfare. Finally, the third section included demographic questions including information on gender, age, college major, country of origin, and current level of study. The full survey is available in the Supplementary material.

**Table 1:** Survey questions from Section 1 assessing students' personal experience and agreement statements from survey Section 2 evaluating the importance students placed on animal and swine welfare topics and their perceived level of confidence in this area in a 5-point Likert scale.

Number	Survey Questions
Section 1 (Multiple choice)	
1	Have you ever taken a welfare course before?
2	How often do you interact with pigs?
3	Where have you worked with or interacted with pigs the most?
4	Do you plan to work with pigs once you graduate?
5	If you had questions about pig welfare, what primary resource(s) would you use?
Section 2 (Statements- Likert scale)	
6	Animal welfare is important to me.
7	Learning about pig welfare is important for my professional education.
8	I feel confident talking about pig welfare and sharing my informed opinion with others.
9	If faced with pig welfare problems on-farm, I would feel confident in addressing these problems using my previous knowledge and experience.
10	How important do you consider the following aspects related to pig welfare? (Health, production, behavior, pain, emotional states, and ethics/societal concerns)

### 2.3 Statistical analysis

All analyses were conducted using Stata/IC 17 (StataCorp LP, College Station, Texas). Data was initially checked for missing values and recording errors. Responses to questions that were left blank or in which participants opted not to disclose information, were considered missing data. Descriptive analyses for numerical variables (mean, standard deviation, and range), as well as frequency (%) for categorical variables, were calculated to describe participant demographics and their personal experiences with swine.

Spearman's correlation was used to explore the strength and direction of the relationship between 1) students' confidence in addressing pig welfare problems on-farm, 2) their confidence in discussing and sharing their informed opinion on pig welfare, 3) their perceived importance on learning about pig welfare, 4) the importance of animal welfare, and 5) various aspects related to animal welfare (health, production, behavior, pain, emotional states, and ethics/societal concerns for pig welfare). Correlations were considered very strong (*r* = ≥0.90) strong (*r* = 0.70–0.89), moderate (*r* = 0.40–0.69), weak (*r* = 0.10–0.39) and negligible (*r* = 0.00–0.10) based on categories described by Schober et al. (25).

Cluster analysis was used as a tool for categorizing study participants into groups that were similar in regard to their survey responses regarding attitudes and importance placed on swine and animal welfare and their confidence in their training to address and discuss swine welfare issues in order to address this study's objectives (26–28). The analysis used complete linkage hierarchical clustering, which defines the distance between clusters by the farthest distance between any two individuals in different clusters. The similarity between individuals was calculated using standard Euclidean (L2) distances. Analysis was performed using four variables (Q6–Q9; Table 1) to capture student perceptions of the importance of animal and swine welfare and their perceived level of confidence in addressing and discussing animal and swine welfare.

For each cluster, a separate multivariable logistic regression model was created in order to investigate the effect of predictors of interest on the odds of participants being part of each cluster. The predictors of interest included age, gender, country of residence, college major, current level of study, students' plan to work with pigs in the future, completion of animal welfare course, frequency of interactions with pigs, setting where students have interacted with pigs the most, and primary resources used when having questions regarding pig welfare.

Model-building steps included first verifying that every category within each predictor of interest had a sufficient sample size to be considered in the model. Four predictor variables required recategorization. Student's frequency of interactions with pigs was classified into three categories, with daily, weekly, and monthly grouped into a single regular interactions category (1= Regular interactions; 2= Yearly; and 3= Never). Country of residence was categorized into three groups, with Brazil and Ecuador retained as separate categories and all the remaining countries combined into other countries category (1= Brazil; 2= Ecuador; 3= Other countries). Gender was classified into two categories (1= Female and 2= Male), with the non-binary set as missing. The setting where students have interacted with pigs the most was categorized into two groups, with internship or externship and university classes/labs combined into a single formal experience category and the category “other” set to missing (1= Formal experience; 2= Own Experience).

Secondly, univariate logistic models were built and a conservative *P* value of < 0.2 was used for screening variables that moved into the full final model. Finally, multivariable models were built using a backward stepwise approach, and final statistical significance was declared at *P* < 0.05 (29).

## 3 Results

A total of 312 surveys were received from Ecuador, Brazil, Chile, and Honduras, as well as three students from one of the above identified countries that were currently residing in the United States at the time of survey completion. From the received surveys, 96 were excluded because they were completely unanswered, leaving a total of 216 usable participant surveys for analysis.

### 3.1 Demographics and Spearman's correlations

Descriptive statistics for survey participant demographics and their experience working with pigs are summarized in Table 2. No strong correlations were found between the variables using Spearman's correlation and have not been included in the results (Supplementary Table A).

**Table 2:** Descriptive statistics on demographic information and students' experience working with pigs.

Variable	Number
Age, in years	
Average	21
Range	17-46
Gender, % (*n*)	
Female	70.0 (147)
Male	29.5 (62)
Non-binary	0.5 (1)
College major, % (*n*)	
Veterinary medicine	86.2 (181)
Veterinary medicine and animal science	7.6 (16)
Animal science	4.8 (10)
Other (Agronomy and food science)	1.4 (3)
Current country of residence, % (*n*)	
Ecuador	82.4 (178)
Brazil	8.8 (19)
Chile	5.1 (11)
Honduras	2.3 (5)
USA	1.4 (3)
Current level of study, % (*n*)	
Undergraduate	94.2 (196)
Graduate	5.8 (12)
Taken a welfare course before, % (*n*)	
Yes	50.2 (108)
No	49.8 (107)
Plan to work with pigs in the future, % (*n*)	
No	26.2 (56)
Yes	16.8 (36)
I am not sure	57.0 (122)
Frequency of pig interaction, % (*n*)	
Daily	2.3 (5)
Weekly	2.3 (5)
Monthly	11.2 (24)
Yearly	37.2 (80)
Never	47.0 (101)
Interacted or worked with pigs the most, % (*n*)	
At university classes/Labs	33.3 (38)
Internship or externship	14.0 (16)
Own experience (personal farm, friends' farm, etc.)	48.3 (55)
Other (trips, university clubs, and agricultural high school)	4.4 (5)

### 3.2 Cluster analysis

**Figure 1:**
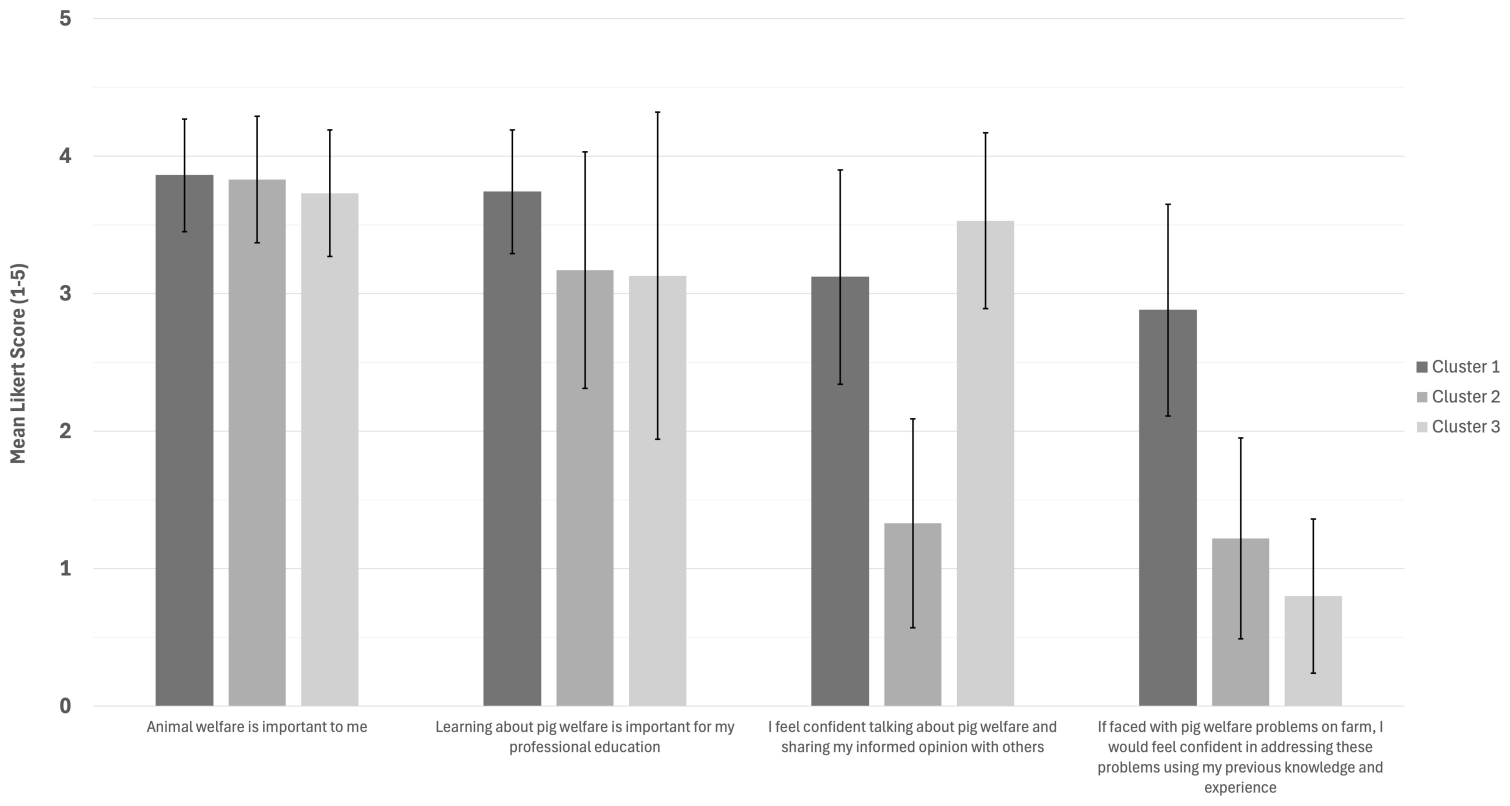
Cluster agreement for survey statements outcomes (mean + SD) on a 5-point Likert scale (1= strongly disagree, 2 = disagree, 3 = neither agree nor disagree, 4 = agree, and 5 = strongly agree).

Within this study, three distinct clusters were identified (Figure 1).

#### 3.2.1 Cluster 1—High welfare and swine education importance, neutral communication and problem-solving confidence

Cluster 1 consisted of 139 participants (65.6% of the total) and represented the majority of the survey respondents. Students belonging to this cluster considered animal welfare as important (3.86; SD = 0.41) and valued learning about swine welfare for their professional education (3.74; SD = 0.45). They reported neutral levels of confidence in talking about and sharing their opinion on pig welfare (3.12; SD = 0.78) and addressing pig welfare problems on farms based on their previous knowledge and experience (2.88; SD = 0.77).

#### 3.2.2 Cluster 2—High welfare and neutral swine welfare education importance, low communication and problem-solving confidence

Cluster 2 consisted of 58 participants (27.3% of the total). Although participants belonging to this group considered animal welfare important (3.83; SD = 0.46), their agreement on the value of learning about swine welfare for their professional education was neutral (3.17; SD = 0.86). Additionally, students belonging to this cluster were not confident in talking about or sharing their opinion on swine welfare (1.33; SD = 0.76), nor addressing pig welfare problems on farms based on their previous knowledge and experience (1.22; SD = 0.73).

#### 3.2.3 Cluster 3—High welfare and neutral swine welfare education importance, high communication and low problem-solving confidence

Lastly, Cluster 3 consisted of 15 participants (7.1% of the total) representing the minority of survey respondents. Cluster 3 was characterized by students who considered animal welfare as important (3.73; SD = 0.46) but were neutral in their agreement on the importance of learning about swine welfare for their professional education (3.13; SD = 0.77). Students in this cluster had confidence in talking about or sharing their opinion on swine welfare (3.53; SD = 0.64) but were the least confident in addressing welfare problems on farms based on their previous knowledge and experience (0.80; SD = 0.56; Figure 1).

### 3.3 Logistic regression models

The final logistic regression models are presented in Table 3. The analysis showed that “source of information” was the only variable associated with cluster membership. This association was observed only for Clusters 1 and 2. In this regard, participants who reportedly used more information from “Animal charities and advocacy groups” (OR = 2.79, *p* = 0.002) were more likely to belong to Cluster 1. On the other hand, participants that reported obtaining their information on swine welfare from “Swine, livestock, and veterinary associations” (OR = 3.09, *p* = 0.006) and less information from “Animal charities or advocacy groups” (OR = 0.30, *p* = 0.003) were more likely to belong to Cluster 2.

**Table 3:** Final logistic regression model for cluster analysis.

Variable	Category	OR	SE	95% CI	*P-*value
Cluster 1					
Source of information	Animal charities and animal advocacy groups	2.79	0.92	1.46 - 5.33	0.002
Cluster 2					
Source of information	Swine, livestock and veterinary associations	3.09	1.26	1.39 - 6.87	0.006
	Animal charities and animal advocacy groups	0.30	0.12	0.14 - 0.67	0.003

OR, Odd ratio; SE, Standard error; CI, Confidence Interval.

## 4 Discussion

Students in our study were primarily female, young (mean age of 21 ± 4.1), and had limited to no experience working with swine consistently. The population from this study mirrors more general populations of veterinary and animal science students both in Latin America (21) and abroad (1, 2, 24, 30, 31). As more than 80% of participants had never interacted with pigs or interactions had only occurred on a yearly basis, the findings of this study should be interpreted in the context of students with limited exposure to swine. Although limited interaction with pigs has been commonly reported in swine welfare studies (30–32), prior experience with swine has been shown to influence student awareness and attitudes (30, 33, 34). Therefore, future work should consider how the frequency of student's exposure to swine influences individual perception of animal welfare issues in Latin America.

Additionally, a quarter of the students participating in this study do not intend to work with pigs in the future and more than half of participating students were unsure if they would work with pigs following graduation. These results are important as they provide an insight into swine welfare perceptions in animal professional communities that will likely work outside of the swine industry. For example, although companion animal veterinarians do not typically address animal welfare issues on commercial swine farms, these professionals do play an essential role in safeguarding animal welfare by serving in positions that guide public policy, research, and education (10, 35). This can be demonstrated in working groups developed by the American Veterinary Medical Association (AVMA) that bring together veterinarians with varying specialties to provide input on important animal welfare guidelines like depopulation and euthanasia (36). Thus, even if these participants do not intend to work directly with pig production upon graduation, they might serve as important allies and leaders in the veterinary and animal science community, potentially shaping the future of pig welfare in Latin America.

In addition to exploring demographic factors, cluster analysis was also conducted to assess students' views on the importance of animal welfare and swine education, as well as their perceived confidence in discussing and addressing swine welfare issues. The cluster analysis revealed three distinct clusters among participating students. In general, across all clusters, students had a strong consensus that animal welfare is important. In this sense, the results from this study are consistent with previous research conducted on student populations in North America (24, 37), Europe (38), and other Latin American countries (19, 22, 23). For instance, a study conducted in Chile stated that over 97% of students across two different universities and careers perceived that animal welfare is important in production systems (22). Similar percentages were also documented in animal science student populations in the United States (97%) (24). Our findings reflect and confirm the importance of animal welfare as a social issue in Latin America, as well as the relevance this topic has for future animal professionals in this region.

Despite this general consensus on the importance of animal welfare, students' perceptions and attitudes regarding swine welfare as part of their professional education varied. Cluster 1 demonstrated a higher numerical level of perceived importance of swine welfare for their professional education, while Clusters 2 and 3 remained closer to neutral on this subject neither acknowledging nor rejecting the value of implementing this information into their curriculum. Although these differences are subtle, it should be noted that one in four participants did not recognize the value swine welfare education has for their professional training. This contrasts with surveyed student populations elsewhere, where future animal professionals agreed that animal welfare education specific to farm animals is important (2) and relevant to their professional training (37). This issue becomes more relevant as university coursework in swine welfare continues to grow in Latin America (17), suggesting that increased commitment to swine welfare in higher education has not yet resonated completely with students' perceptions and value toward this topic. This concern is reinforced within our study population, as half of the students had completed an animal welfare course, yet course completion did not appear to influence participants' perceptions of the importance of swine welfare. Evidence regarding the influence of animal welfare coursework on perceptions is inconsistent in the scientific literature, with some studies supporting it can influence students' views and attitudes (39, 40), while others report no such effect (41, 42). Our results are consistent with the latter, suggesting limited influence of animal welfare coursework. This may indicate that students' attitudes and perspectives might already be established before formal training or be more strongly influenced by individual or personal values (41, 42). As such, these findings highlight the need to understand the underlying drivers of this issue and to ensure that courses offered by Latin American universities are not only available to students, but also denote quantifiable impact on student knowledge and awareness of the importance of this topic.

Lastly, this study sought to explore students' confidence in discussing and addressing welfare issues specific to pigs. Cluster 1 reported neutral confidence in both discussing and addressing swine welfare on farm while Cluster 2 reported low confidence in both aspects. A potential contributor to the observed differences between Cluster 1 and 2 may be associated with results captured in the logistic regression analysis given information sources used when addressing questions specific to swine welfare were a significant factor contributing to cluster membership. Students belonging to Cluster 1 relied more significantly on animal welfare information acquired from animal charities and advocacy groups, while students who were members of Cluster 2 relied less on these sources and more on information obtained from swine, livestock, and veterinary associations. Although no studies to date have explored animal welfare confidence levels with information sources, previous work by Mckendree et al. (43) explored the importance of information sources on perceptions and priority of animal welfare issues. The 2014 study suggested that individuals that already had high levels of prioritization of animal welfare issues tended to identify animal advocacy and charities as their primary information source. In addition, this study also acknowledged that participants believed that information sources from animal charities and advocacy groups were easier to access and utilized more attractive websites to explain welfare issues. Although it is unclear what direct role varying information sources have on student confidence in discussing and addressing swine welfare issues, future work is needed to further explore this area of research and determine what factors within these information sources drive changes in student confidence and knowledge.

In contrast to Cluster 1 and 2, a minority group of students belonging to Cluster 3 reported feeling confident in discussing swine welfare, even though they were the least confident in addressing welfare issues on farms. Confidence in one's own ability, also termed self-efficacy, refers to one's perceived capabilities to learn or perform actions at designated levels (44). Self-efficacy is a concept that has been widely used to evaluate and inform educational programs and training in veterinary careers, as it has direct implications on education, motivation, and future professional behavior (45–47). However, because self-efficacy is a self-reported measure, it can be influenced by many factors that could lead students to overestimate their competence (48), which may help explain why a minority of students in Cluster 3 reported high confidence in discussing pig welfare but low confidence in addressing those issues on farm.

Overall, our findings indicate that most students do not feel fully prepared to discuss or resolve swine welfare challenges. This issue might be further explained by students' lack of exposure to pigs, as previous research has shown that hands-on experiences can improve students' knowledge and confidence with swine welfare (30, 32). As most participants in our study had limited or no previous interaction with pigs, this may have contributed to low confidence levels observed across clusters. Future studies should evaluate whether increasing practical exposure to pigs improves Latin American student' confidence in discussing and addressing swine welfare issues. Ultimately, universities in the region should aim to develop educational practices that better prepare students to discuss and resolve swine welfare challenges, given the importance this group of students has for the future of swine welfare in the region.

## 5 Limitations

Despite our study results being a valuable contribution to an understudied population in the animal welfare educational scientific literature and a pioneer in exploring swine welfare perspectives from veterinary and animal science students in Latin America, some limitations should be acknowledged.

First, the survey was distributed through various personal institutional contacts in Latin America with the aim of capturing a broad range of student perspectives across a vast geographical and diverse region. These contacts further shared the survey link within their networks to reach a broader audience and expand the sample size. Thus, calculating a response rate was not feasible, as the total number of students who ultimately received the survey couldn't be determined. In addition, most of our respondents were from Ecuador, highlighting the need for greater representation from other countries in future studies. Therefore, care must be taken when generalizing these findings to a broader population.

Additionally, self-administered questionnaires with optional questions may introduce the possibility of selection bias, where students who already have an interest in animal welfare may be more inclined to participate (1). This could also limit transferability to other populations and disciplines due to geographical, gender, and profession bias. Despite these limitations, our study provides a foundational base for future research into this region where subsequent studies could be built upon to expand the knowledge of swine welfare and its importance in higher education in Latin America.

## 6 Conclusions

The findings from this study provide meaningful insights into students' perspectives and views on swine welfare, as well as offer a better understanding of how confident they feel in communicating and addressing pig welfare issues. Our results showed consistent agreement among students on the importance of animal welfare. However, the findings also revealed that not all students consider swine welfare education important, despite universities in the region increasingly incorporating swine welfare topics in their coursework. This highlights a misalignment of academic offering, and the competencies students believe as important for their professional training. Therefore, Latin American universities should prioritize research to uncover the root of this issue, as well as support evidence-based curricular reforms to improve students' views on the importance of this topic in order to provide a well-rounded education. Additionally, students' perceptions and confidence to discuss pig welfare issues seem to be influenced by the source of information they rely on. In the future, more research studies should further explore this factor to assess the extent to which it influences student perceptions and confidence. Finally, participants generally do not feel fully prepared to communicate nor address swine welfare challenges, which calls the need for universities to start developing and incorporating educational strategies that help to mitigate these issues and tackle this gap.

## Data Availability

The datasets generated and analyzed in this study are not publicly available due to ethical and confidentiality constraints, as participants consented to restricted use of their data by the research team and the study protocol. Requests to access the datasets should be directed to Gustavo Donoso, gadonoso@ncsu.edu.

## References

[1] Beaver A Ventura BA. Undergraduate student attitudes toward animal welfare science: an investigation to inform teaching approaches. Anim Welf. (2025) 34:e58. doi: 10.1017/awf.2025.10032 40988864 PMC12451403

[2] Mijares S Sullivan P Cramer C Román-Muñiz N Edwards-Callaway L. Perceptions of animal welfare and animal welfare curricula offered for undergraduate and graduate students in animal science departments in the United States. Transl Anim Sci. (2021) 5:txab222. doi: 10.1093/tas/txab222 35036856 PMC8755489

[3] Broom DM. Animal welfare: an aspect of care, sustainability, and food quality required by the public. J Vet Med Educ. (2010) 37:83–8. doi: 10.3138/jvme.37.1.83 20378884

[4] Association of American Veterinary Medical Colleges. Competency-Based Veterinary Education (CBVE): Framework 2.0. Washington: AAVMC (2024).

[5] The Royal College of Veterinary Surgeons. The Royal College of Veterinary Surgeons Day One Competences. London: The Royal College of Veterinary Surgeons (2022).

[6] Huertas SM Gallo C Galindo F. Drivers of animal welfare policy in the Americas. Rev Sci Tech Off Int Epiz. (2014) 33:67–76. doi: 10.20506/rst.33.1.2255 25000778

[7] Law J. Swine veterinarians—Key players in the pork production chain. Can Vet J. (2021) 62:515–6. 33967293 PMC8048199

[8] Kipperman BS. The role of the veterinary profession in promoting animal welfare. J Am Vet Med Assoc. (2015) 246:502–4. doi: 10.2460/javma.246.5.502 25671279

[9] Patterson G Brown JT Almond GW Ramirez A Pittman J Pieters M . Challenges and opportunities in modern swine veterinary education. J Am Vet Med Assoc. (2022) 260:711–3. doi: 10.2460/javma.21.10.0443 35239507

[10] Littlewood KE Beausoleil NJ. Two domains to five: advancing veterinary duty of care to fulfill public expectations of animal welfare expertise. Animals. (2021) 11:3504. doi: 10.3390/ani11123504 34944280 PMC8698054

[11] Vargas-Bello-Pérez E Miranda-de la Lama GC Teixeira DL Enríquez-Hidalgo D Tadich T Lensink J. Farm animal welfare influences on markets and consumer attitudes in Latin America: the cases of Mexico, Chile and Brazil. J Agric Environ Ethics. (2017) 30:697–713. doi: 10.1007/s10806-017-9695-2

[12] Roppa L Duarte ME Kim SW. Pig production in Latin America. Anim Biosci. (2024) 37:786–93. doi: 10.5713/ab.23.0453 38419541 PMC11016694

[13] Gallo C Tadich N Huertas S César D Da Costa MP Broom DM. Animal welfare education in Latin América. In: Proceedings of the International Conference on Animal Welfare Education: Everyone Is Responsible, Brussels, Belgium (2010). p. 1–2.

[14] Molento CFM Calderon N. Essential directions for teaching animal welfare in South America: -EN- -FR- Principales orientations pour l'enseignement du bien-être animal en Amérique du Sud -ES- Orientaciones básicas para la enseñanza del bienestar de los animales en América del Sur. Rev Sci Tech. (2009) 28:617–25. doi: 10.20506/rst.28.2.1899 20128472

[15] Tadich NA Molento CFM Gallo CB. Teaching animal welfare in some veterinary schools in Latin America. J Vet Med Educ. (2010) 37:69–73. doi: 10.3138/jvme.37.1.69 20378882

[16] Gallo C Tadich T Huertas S Cesar D Paranhos da. Costa N, Broom D. Animal Welfare Education in Latin America. First Int Conf Anim Welf Educ. (2010).

[17] Mota-Rojas D Orihuela A Strappini-Asteggiano A Nelly Cajiao-Pachón M Agüera-Buendía E Mora-Medina P . Teaching animal welfare in veterinary schools in Latin America. Int J Vet Sci Med. (2018) 6:131–40. doi: 10.1016/j.ijvsm.2018.07.003 30564587 PMC6286393

[18] Gallo C Véjar L Galindo F Huertas SM Tadich T. Animal welfare in Latin America: trends and characteristics of scientific publications. Front Vet Sci. (2022) 18:9. doi: 10.3389/fvets.2022.1030454 PMC9716110 36467645

[19] Valverde A González-Miranda JA Sevilla F Mora S Roldan ERS Vargas C . Perceptions of animal welfare on livestock: evidence from college agronomy students in Costa Rica. Animals. (2024) 14:1398. doi: 10.3390/ani14101398 38791616 PMC11117388

[20] Ceballos-Olvera I Tolentino-García S Luna-Castro S Ruiz-Albarrán M Torres-Rodríguez L Peña-Avelino LY. Work preferences and animal welfare perception of veterinary medicine and animal science students in Northeastern Mexico. J Appl Anim Welf Sci. (2023) 26:80–90. doi: 10.1080/10888705.2021.1925899 33988054

[21] Andrighetto Canozzi ME Cardoso S Foguesatto CR Rossi Borges JA. Perception of Brazilian agricultural sciences students on animal welfare: a profile-based approach. J Appl Anim Welf Sci. (2025) 28:230–42. doi: 10.1080/10888705.2023.2268505 37818850

[22] Vargas-Bello-Pérez E Obermöller-Bustamante C Faber I Tadich T Toro-Mujica P. Knowledge and perception on animal welfare in Chilean undergraduate students with emphasis on dairy cattle. Animals. (2021) 11:1921. doi: 10.3390/ani11071921 34203442 PMC8300360

[23] Monsalve S de Souza PV Lopes AS Leite LO Polo G Garcia R. Medicina veterinaria forense, bienestar y maltrato animal: percepciones y conocimiento de estudiantes de medicina veterinaria Colombianos y Brasileños. J Vet Med Educ. (2021) 48:764–73. doi: 10.3138/jvme-2019-0138.es 34898396

[24] Sullivan P Mijares S Davis M Oselinsky K Cramer C Román-Muñiz N . A nationwide survey of animal science students' perceptions of animal welfare across different animal categories at institutions in the United States. Animals. (2022) 12:2294. doi: 10.3390/ani12172294 36078014 PMC9454941

[25] Schober P Boer C Schwarte LA. Correlation coefficients: appropriate use and interpretation. Anesth Analg. (2018) 126:1763. doi: 10.1213/ANE.0000000000002864 29481436

[26] Viana GGF Arruda AG Rossi GAM. Assessing food safety practices and foodborne illness risk factors in Brazilian households. PLoS ONE. (2025) 20:e0325070. doi: 10.1371/journal.pone.0325070 40531919 PMC12176236

[27] Alves LKSKS Pairis-Garcia MD Arruda AGG de Melo CAF Gomes N de AC Hoshino RY . Perceptions of swine euthanasia among Brazilian caretakers from non-integrated swine farms. Front Vet Sci. (2025) 6:11. doi: 10.3389/fvets.2024.1513141 PMC11743181 39834925

[28] Duda RO. Pattern Classification (2nd edn). New York: Wiley (2001).

[29] Pfeiffer D. Veterinary Epidemiology: An Introduction. Chichester: John Wiley & Sons (2010). p 151.

[30] Ventura BA Terreaux CMHA Zhitnitskiy PE. Veterinary student knowledge and attitudes about swine change after lectures and a farm visit. J Vet Med Educ. (2021) 48:492–502. doi: 10.3138/jvme-2019-0160 33226905

[31] Proudfoot KL Améndola L Pairis-Garcia M Hernandez MH Ventura B Millman S. Veterinary student knowledge, attitudes, and perspectives on differing viewpoints regarding pain management after a role-play case study on piglet castration. J Vet Med Educ. (2025) 17:e20240161. doi: 10.3138/jvme-2024-0161 40549547

[32] Wright AJ Powney SL Nevel A Wathes CM. Pig welfare assessment: development of a protocol and its use by veterinary undergraduates. J Vet Med Educ. (2009) 36:50–61. doi: 10.3138/jvme.36.1.50 19435990

[33] Cavalieri J. Veterinary student responses to learning activities that enhance confidence and ability in pig handling. J Vet Med Educ. (2009) 36:39–49. doi: 10.3138/jvme.36.1.39 19435989

[34] Tawse J. Consumer attitudes toward farm animals and their welfare: a pig production case study. Biosci Horiz Int J Stud Res. (2010) 3:156–65. doi: 10.1093/biohorizons/hzq020

[35] AVMA. Joint AVMA-FVE-CVMA Statement on the Roles of Veterinarians in Promoting Animal Welfare (2020). Available online at: https://www.avma.org/resources-tools/avma-policies/joint-avma-fve-cvma-roles-veterinarians-promoting-animal-welfare (Accessed April 27, 2026).

[36] Leary S Anthony R Gwaltney-Brant S Pritchett-Corning K Ramirez A. AVMA Guidelines for the Depopulation of Animals: 2026 Edition. Schaumburg: American Veterinary Medical Association (2026).

[37] Anderson NC Underwood L Byrd CJ. Student reported learning of swine and dairy welfare concepts following a virtual reality livestock farm experience. J Appl Anim Welf Sci. (2025) 0:1–13. doi: 10.1080/10888705.2025.2559700 40955860

[38] Anneberg I Lassen J Sandøe P. For the sake of production—and the animal, and me. How students at Danish agricultural colleges perceive animal welfare. Animals. (2021) 11:696. doi: 10.3390/ani11030696 33807571 PMC8001740

[39] Contreras ET Vanderstichel R. Enhancing veterinary education through a novel animal welfare and behavior course at a new veterinary university. J Vet Med Educ. (2026) 53:223–35. doi: 10.3138/jvme-2024-0108 39951258

[40] Hazel SJ Signal TD Taylor N. Can teaching veterinary and animal-science students about animal welfare affect their attitude toward animals and human-related empathy? J Vet Med Educ. (2011) 38:74–83. doi: 10.3138/jvme.38.1.74 21805938

[41] Çavuşoglu E Uzabaci E. Attitudes of Turkish veterinary students toward farm animal welfare. Anim Welf. (2021) 30:325–30. doi: 10.7120/09627286.30.3.010

[42] Robbins JA Danielson JA Johnson AK Parsons RL Jorgensen MW Millman ST. Attitudes toward animals and belief in animal mind among first-year veterinary students before and after an introductory animal welfare course. Anim Welf. (2021) 30:409–18. doi: 10.7120/09627286.30.4.004

[43] McKendree MGS Croney CC Widmar NJO. Effects of demographic factors and information sources on United States consumer perceptions of animal welfare. J Anim Sci. (2014) 92:3161–73. doi: 10.2527/jas.2014-6874 24962533

[44] Schunk DH DiBenedetto MK. “Self-efficacy and human motivation,” in *Advances in Motivation Science*, Vol. 8, Amsterdam: Elsevier (2021). pp. 153–179. doi: 10.1016/bs.adms.2020.10.001

[45] Morin DE Royster E Johnson-Walker YJ Molgaard L Fetrow J. Effects of an 8-week dairy production medicine course on veterinary student self-confidence and perceptions of knowledge and skills used by dairy veterinarians. J Vet Med Educ. (2020) 47:290–306. doi: 10.3138/jvme.1117-165r 32486943

[46] Jensen KC Kleinsorgen C Karbe GT. Evaluation of knowledge, self-assessment of skills and self-perception in the role of small animal practitioner of veterinary students before and after a structured clinical rotation. Vet Sci. (2026) 13:113. doi: 10.3390/vetsci13020113 41745907 PMC12945202

[47] Abildgaard M Kron M Carlund T Enlund KB. Attitudes, experience, and self-confidence of veterinary and veterinary nursing students in small animal dentistry: a survey study. J Vet Med Educ. (2025) 52:483–92. doi: 10.3138/jvme-2023-0183 39504221

[48] Peffer PAL Davis ME. Self-estimates of performance in animal sciences courses and factors that influence perceived competence. NACTA J. 2018 62:122–9. Available online at: https://www.jstor.org/stable/90022546

