# Supplementary material for: Swine welfare perceptions among future animal professionals in Latin America
Source: Front Vet Sci. 2026 Jul 23;13:1874259. doi: 10.3389/fvets.2026.1874259 (PMC13441914; doi:10.3389/fvets.2026.1874259)
Supplement: Supplementary file 1 [file Table_1.DOCX]

Supplementary Materials:

1. Student Survey Questions

Section 1: Students personal experience with pigs

Q1 Have you ever taken a welfare course before?

- Yes (1)
- No (2)

Q2 How often do you interact with pigs?

- Daily (1)
- Weekly (2)
- Monthly (3)
- Yearly (4)
- Never (6)

Display this question:

If How often do you interact with pigs? != Never

Q3 Where have you worked with or interacted with pigs the most?

- At university classes/Labs (1)
- Internship or externship (2)
- Own experience (personal farm, employee profession, friends, etc.) (3)
- Other (4) __________________________________________________

Q4 Do you plan to work with pigs once you graduate?

- Yes (1)
- No (2)
- I am not sure (3)

Q5 If you had questions about pig welfare, what primary resource (s) would you use? (Select all that apply)

- Peer-reviewed publications (1)
- Swine and livestock/veterinary associations (2)
- Professors and industry professionals (3)
- Animal charities and animal advocacy groups (4)
- Social media (Instagram, X, Facebook, etc.) (5)
- Peers (6)
- Relatives (7)
- Other (8) __________________________________________________

**Section 2: Importance placed on animal and swine welfare topics and level of confidence**

Q6 Animal welfare is important to me.

- Strongly agree (1)
- Somewhat agree (2)
- Neither agree nor disagree (3)
- Somewhat disagree (4)
- Strongly disagree (5)

Q7 Learning about pig welfare is important for my professional education.

- Strongly agree (1)
- Somewhat agree (2)
- Neither agree nor disagree (3)
- Somewhat disagree (4)
- Strongly disagree (5)

Q8 I feel confident talking about pig welfare and sharing my informed opinion with others.

- Strongly agree (1)
- Somewhat agree (2)
- Neither agree nor disagree (3)
- Somewhat disagree (4)
- Strongly disagree (5)

Q9 If faced with pig welfare problems on farm, I would feel confident in addressing these problems using my previous knowledge and experience.

- Strongly agree (1)
- Somewhat agree (2)
- Neither agree nor disagree (3)
- Somewhat disagree (4)
- Strongly disagree (5)

Q10 How important do you consider the following aspects related to pig welfare?

|  | Extremely important (1) | Very important (2) | Moderately important (3) | Not very important (4) | Not important at all (5) | I don't know (6) |
| --- | --- | --- | --- | --- | --- | --- |
| Health (e.g. disease, preventative care) (1) |  |  |  |  |  |  |
| Production (e.g. increased mortality rise, poor growth) (2) |  |  |  |  |  |  |
| Behavior (e.g. positive interactions with caretakers, access to environmental enrichment) (3) |  |  |  |  |  |  |
| Pain (e.g. providing pain relief during painful procedures) (4) |  |  |  |  |  |  |
| Emotional States (e.g. fear free handling, a peaceful death) (5) |  |  |  |  |  |  |
| Ethics/ societal concerns (e.g. creation of laws and regulations, environmental sustainability) (6) |  |  |  |  |  |  |

Section 3: Demographics

Q11 Gender

- Male (1)
- Female (2)
- Other (4) __________________________________________________

Q12 Age (years)

________________________________________________________________

Q13 College major

- Veterinary medicine (1)
- Animal science (2)
- Another (3) __________________________________________________

Q14 Current level of study

- Undergraduate (1)
- Graduate (2)
- Veterinary (3)

**Table A.** Spearman correlation for confidence in addressing pig welfare problems on farm based on their prior knowledge and experience, their confidence in discussing pig welfare and sharing their informed opinion with others, the importance they place on various aspects animal welfare and learning about pig welfare

|  | Confident addressing pig welfare on farm | Confident discussing pig welfare | Importance of pig welfare education | Animal welfare importance | Importance placed on Health | Importance placed on Production | Importance placed on Behavior | Importance placed on Pain | Importance placed on Emotion | Importance placed on Ethics and Societal concerns |
| --- | --- | --- | --- | --- | --- | --- | --- | --- | --- | --- |
| Confident addressing pig welfare on farm | 1.0000 |  |  |  |  |  |  |  |  |  |
| Confident discussing pig welfare | 0.5120 | 1.0000 |  |  |  |  |  |  |  |  |
| Importance of pig welfare education | 0.1789 | 0.3137 | 1.0000 |  |  |  |  |  |  |  |
| Animal welfare importance | 0.0846 | 0.1396 | 0.3444 | 1.0000 |  |  |  |  |  |  |
| Importance placed on Health | 0.0078 | 0.1102 | 0.3667 | 0.3435 | 1.0000 |  |  |  |  |  |
| Importance placed on Production | 0.2312 | 0.1830 | 0.2861 | 0.0856 | 0.3111 | 1.0000 |  |  |  |  |
| Importance placed on Behavior | 0.0638 | 0.0980 | 0.3273 | 0.2368 | 0.4082 | 0.2636 | 1.0000 |  |  |  |
| Importance placed on Pain | 0.1103 | 0.0224 | 0.1810 | 0.3106 | 0.4244 | 0.1665 | 0.4074 | 1.0000 |  |  |
| Importance placed on Emotion | 0.0826 | 0.0957 | 0.3152 | 0.3433 | 0.3942 | 0.1769 | 0.4438 | 0.4953 | 1.0000 |  |
| Importance placed on Ethics and Societal concerns | 0.0868 | 0.0095 | 0.1741 | 0.2011 | 0.3596 | 0.1395 | 0.4430 | 0.3923 | 0.4665 | 1.0000 |
